# Predicting pathological grade of stage I pulmonary adenocarcinoma: a CT radiomics approach

**DOI:** 10.3389/fonc.2024.1406166

**Published:** 2024-09-27

**Authors:** Xiaoni Huang, Yang Xue, Bing Deng, Jun Chen, Jiani Zou, Huibin Tan, Yuanliang Jiang, Wencai Huang

**Affiliations:** ^1^ The First School of Clinical Medicine, Southern Medical University, Guangzhou, China; ^2^ Department of Radiology, General Hospital of Central Theater Command of the People’s Liberation Army, Wuhan, China; ^3^ Wuhan University of Science and Technology School of Medicine, Wuhan, China; ^4^ Radiology Department, Bayer Healthcare, Wuhan, China

**Keywords:** tomography, X-ray computed, adenocarcinoma of lung, neoplasm grading, logistic models, nomograms

## Abstract

**Objectives:**

To investigate the value of CT radiomics combined with radiological features in predicting pathological grade of stage I invasive pulmonary adenocarcinoma (IPA) based on the International Association for the Study of Lung Cancer (IASLC) new grading system.

**Methods:**

The preoperative CT images and clinical information of 294 patients with stage I IPA were retrospectively analyzed (159 training set; 69 validation set; 66 test set). Referring to the IASLC new grading system, patients were divided into a low/intermediate-grade group and a high-grade group. Radiomic features were selected by using the least absolute shrinkage and selection operator (LASSO), the logistic regression (LR) classifier was used to establish radiomics model (RM), clinical-radiological features model (CRM) and combined rad-score with radiological features model (CRRM), and visualized CRRM by nomogram. The area under the curve (AUC) of the receiver operating characteristic (ROC) curve and calibration curve were used to evaluate the performance and fitness of models.

**Results:**

In the training set, RM, CRM, and CRRM achieved AUCs of 0.825 [95% CI (0.735-0.916)], 0.849 [95% CI (0.772-0.925)], and 0.888 [95% CI (0.819-0.957)], respectively. For the validation set, the AUCs were 0.879 [95% CI (0.734-1.000)], 0.888 [95% CI (0.794-0.982)], and 0.922 [95% CI (0.835-1.000)], and for the test set, the AUCs were 0.814 [95% CI (0.674-0.954)], 0.849 [95% CI (0.750-0.948)], and 0.860 [95% CI (0.755-0.964)] for RM, CRM, and CRRM, respectively.

**Conclusion:**

All three models performed well in predicting pathological grade, especially the combined model, showing CT radiomics combined with radiological features had the potential to distinguish the pathological grade of early-stage IPA.

## Introduction

In 2020, the International Association for the Study of Lung Cancer (IASLC) proposed a new pathological grading system based on the predominant histopathological subtypes and 20% high-grade pattern of invasive pulmonary adenocarcinoma (IPA) (1). This system was subsequently adopted by the 2021 WHO Classification of Lung Tumors (2). Prior to this, there was no internationally recognized grading system due to the high heterogeneity of pulmonary adenocarcinoma. The architectural grading system, established in 2011, is a commonly used grading system based on the histologic classification of pulmonary adenocarcinoma according to prognostic stratification (3). Numerous studies have confirmed that the presence of a high-grade pattern, even if not predominant, indicating a poor prognosis of patients (4–6). A limitation of the architectural grading system is that it considers only the one most predominant pattern, which may underestimate the pathological grades of IPA with high-grade patterns but not predominant subtype. Sica’s grading system may provide an improvement over the architectural grading system, as it takes into account the two most predominant patterns (7). In contrast, the IASLC grading system integrates both the most predominant pattern and the proportion of high-grade patterns; any tumor with 20% or more high-grade patterns is classified as high-grade IPA. The primary distinction between the IASLC grading system and the other two systems is its emphasis on the presence of high-grade patterns, establishing this threshold as a significant prognostic factor for recurrence and mortality. Furthermore, the IASLC grading system differentiates complex glandular patterns (cribriform and fused gland) from the traditional acinar subtype, categorizing them as high-grade patterns. Previous studies have shown that these complex glandular patterns are associated with poor prognosis, similar to solid and micropapillary subtypes (8, 9). The IASLC grading system has been validated through large-sample cohort studies conducted in various countries, indicating that this new grading system may more effectively predict patient prognosis, particularly in early-stage IPA (6, 10–14). Therefore, the early identification of the pathological grade of IPA could inform subsequent surveillance strategies, surgical approaches, or adjuvant therapies both prior to and following surgery.

However, the pathological invasiveness and grade of IPA are mainly estimated from completely resected tumor specimens instead of using needle biopsy. Because biopsy usually only takes a part of the tumor tissue, which may not fully reflect the heterogeneity of the tumor (15). Therefore, it is difficult to accurately obtain the histological characteristics of the tumor for patients who do not or cannot have surgery. How to non-invasively evaluate the pathological invasiveness and histopathological grading of tumors by preoperative medical images has become an urgent problem to be solved. Radiomics has been an emerging research field in recent years, which can non-invasively reflect tissues underlying pathological and physiological characteristics by converting digital medical images into mineable data and extracting numerous hidden quantitative information from morphological and functional images in a high-throughput manner. Radiomics have been widely studied in the classification of benign and malignant lung tumors (16), differentiation of different histological types of lung cancer (17), prediction of lung cancer prognosis (18), evaluation of treatment effects (19), prediction of genotypes in pulmonary adenocarcinoma (20), prediction of PD-L1 expression and tumor mutation burden (21) and differentiation of immune pneumonitis from radiation pneumonitis (22). Currently, there are limited reports on the application of radiomics to the IASLC grading system for IPA. Therefore, this study aims to investigate the potential of utilizing preoperative CT radiomics combined with clinical and radiological features to predict the pathological grade of stage I IPA.

## Materials and methods

### Study population

This retrospective study was reviewed by our institutional ethics committee, and the patient’s informed consent was waived (Ethical Approval No. 2020035-1). Patients with stage I IPA confirmed by pathology from January 2017 to July 2023 were collected. The exclusion criteria were as follows: (1) the pathological results were obtained by needle biopsy or without complete clinicopathological information; (2) patients with stage II-IV IPA; (3) patients with minimally invasive adenocarcinoma(MIA), invasive mucinous adenocarcinoma(IMA), and other variants; (4) interventions such as needle biopsy or radiotherapy or chemotherapy before CT scan; (5) patients did not have a CT scan within 2 weeks before surgery; (6) the images were unclear and couldn’t be used for analysis.

**Figure 1:**
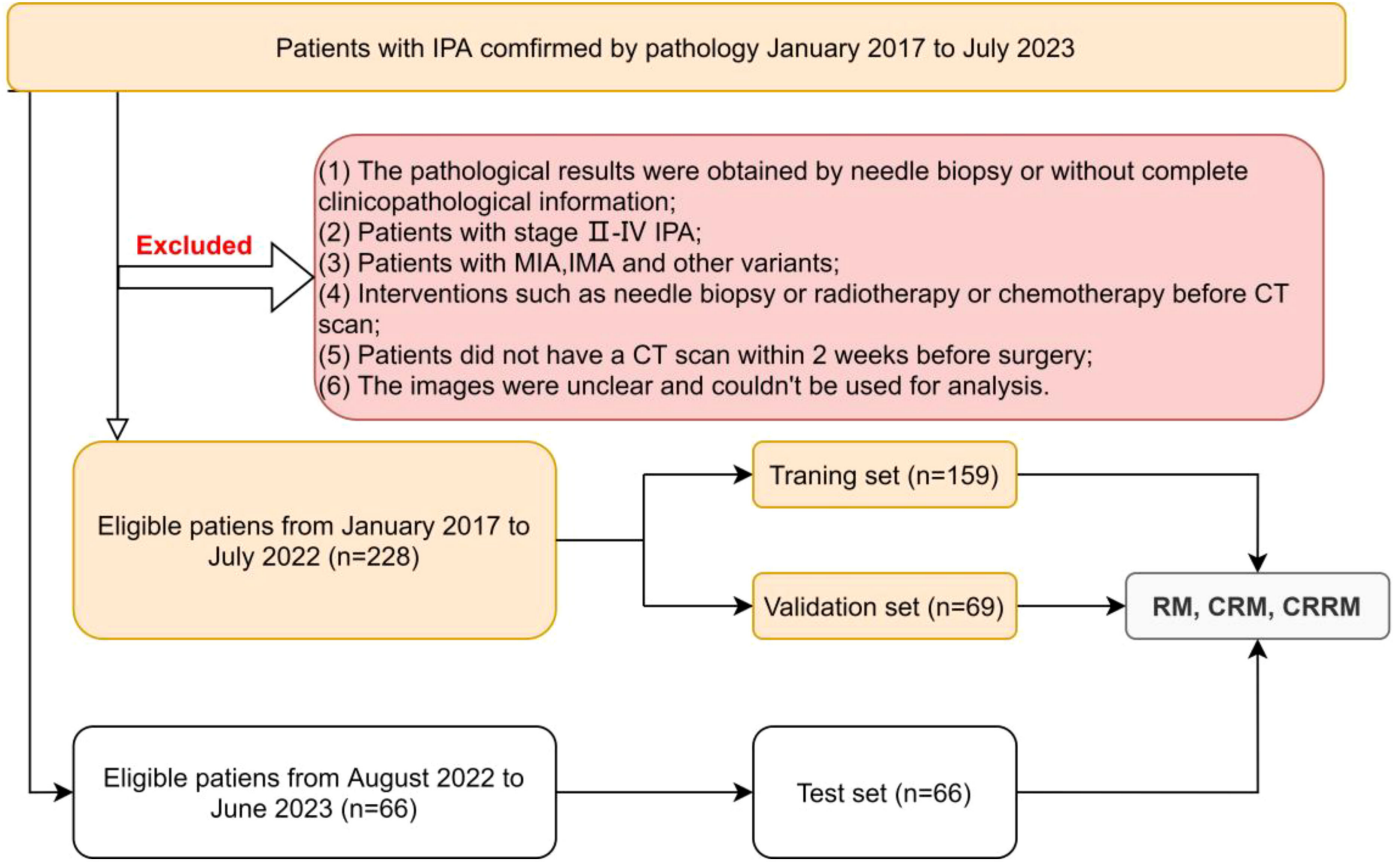
Flowchart of the patient selection.

Finally, a total of 294 eligible patients were included in this study. Among them, 228 patients from January 2017 to July 2022 were randomly divided into the training set and the validation set using a 7:3 random sampling method. Additionally, 66 patients from August 2022 to June 2023 constituted the independent test set. According to the IASLC grading system, patients were divided into low/intermediate-grade and high-grade groups for analysis and discussion (**Figure 1**).

### CT image acquisition

Images were acquired from three different machines.

CT scanner 1: TOSHIBA Aquilion 16-row detector spiral CT, scan parameters: tube voltage 120kV, tube current 250mA, scanning collimation 1.0mm×16, pitch 1.3, rotation time 0.5s/circle, FOV 500mm, acquisition matrix 512×512.

CT scanner 2: GE 16-row detector spiral CT, scan parameters: tube voltage 120kV, tube current changes, scanning collimation 1.0mm×16, pitch 1.375, rotation time 0.5s/circle, FOV 360mm, acquisition matrix 512×512.

CT scanner 3: TOSHIBA Aquilion ONE 320-row detector dynamic volumetric CT, scan parameters: tube voltage 120kV, tube current changes, scanning collimation 0.5mm×84, pitch 1.3, rotation time 0.5s/circle, FOV 400mm, acquisition matrix 512×512.

All scans were performed with breath-hold scanning at the end of deep inspiration, and the scanning range was from the level of the costophrenic angle at the bottom of the lung to the thoracic entrance. 3D high-resolution reconstruction was performed at a sub-workstation after scanning. The reconstructed slice thickness was 2.0 mm. A standard lung window (WL: -550HU, WW: 1600HU) and mediastinal window (WL: 40HU, WW: 320HU) were used for observation.

### Histologic evaluation

Two senior pathologists evaluated pathological sections of postoperative specimens simultaneously, and a consensus diagnosis was reached after discussion. The percentage of each pattern was recorded in 5% increments. All pathological sections were divided into three grades according to the IASLC grading system: low- grade (well-differentiated adenocarcinoma: lepidic predominant tumors with<20% of high-grade patterns), intermediate-grade (moderately differentiated adenocarcinomas, acinar or papillary predominant tumors with<20% of high-grade patterns), and high-grade(poorly differentiated: any tumor with≥20% of high-grade patterns).

### Tumor segmentation and radiomic feature extraction

Tumor segmentation was performed by two radiologists with more than five years’ experience in respiratory system imaging diagnosis using an open-source image processing software-3D Slicer (Version 4.13.0, https://www.slicer.org/). The region of interest (ROI) that covers the entire CT visible tumor was manually contoured slice by slice on the axial plane, avoiding surrounding normal tissues as much as possible. When the nodule was close to the pleura, it was required to contour more than 1 mm from it. Both radiologists were blind to the clinical information and pathological results. The intraclass correlation coefficient (ICC) was used to measure and evaluate inter-observe and test-retest reliability. The features of ICC≥0.75 indicated good repeatability and were reserved for further analysis.

All CT images were resampled using a spline interpolation algorithm to ensure radiographic consistency, and all images were spaced 1mm×1mm×1mm. We extracted radiomic features using PyRadiomics software (https://pyradiomics.readthedocs.io/). A total of 1211 radiomic features were extracted for each patient on CT images. The radiomic features could be classified into seven categories: Shape Features, First Order Features, Gray Level Co-occurrence Matrix (GLCM) Features, Gray Level Dependence Matrix (GLDM) Features, Gray Level Run Length Matrix (GLRLM) Features, Gray Level Size Zone Matrix (GLSZM) Features and Neighborhood Gray-Tone Difference Matrix Features (NGTDM) (23). Three types of images were used to extract these quantitative radiomic features: the Original Image, the Laplacian of Gaussian Image, and the Wavelet Image, which was derived after eight wavelet decompositions. By applying High (H) or Low (L) pass filter in three dimensions, we got eight combinations: LHL, HHL, HLL, HHH, HLH, LHH, LLH, and LLL. A sequence of sigma values was used to generate LoG Image by LoG filter. A high sigma emphasizes coarse textures, while a low sigma emphasizes fine textures. Sigma of 2, 3, 4, and 5 were used in this study.

### Feature selection and construction, validation of radiomic model

Dimensionality reduction for radiomic features was achieved in three steps. Firstly, radiomic features with variance>1.0 were selected. Secondly, Analysis of Variance (ANOVA) was used to choose the statistical influence feature for pathological grade. Lastly, radiomic features were available by selecting pathological grade-related features with non-zero coefficients from the training set using the least absolute shrinkage and selection operation (LASSO) algorithm. With a combination of selected features weighted by their respective coefficients, the rad-score was computed for each patient using the LASSO regression. Both feature selection and radiomic signature construction were performed in the training set. Radiomic signature performance was evaluated using an inter-validation set and independent test set which were not used for model construction.

### Definition of CT Image radiological features

**Figure 2:**
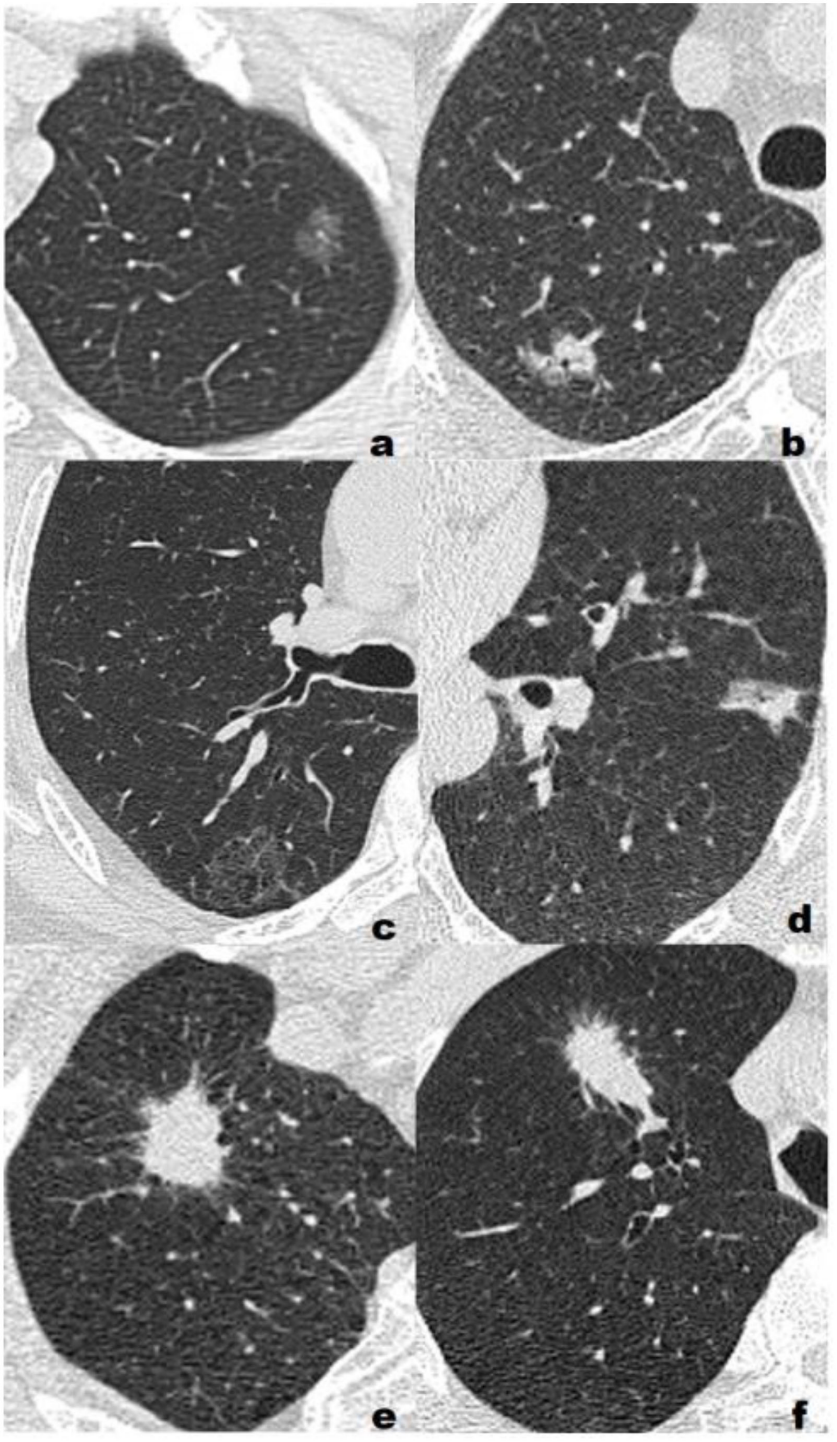
Representative cases of three different pathological grades. **(A)** Low grade, F, 55Y, T1b, a 1.16-cm lepidic predominant adenocarcinoma with no high-grade patterns in the left upper lobe(LUL) manifesting as a pure ground glass nodule(pGGN). All three model classified the lesion as low/intermediate-grade group; **(B)** Low grade, F, 72Y, T1b, a 1.64-cm lepidic predominant adenocarcinoma with 5% high-grade patterns in the right upper lobe(RUL) manifesting as a sub-solid nodule(SSN). All three model classified the lesion as low/intermediate-grade group; **(C)** Intermediate-grade, M, 57Y, T1c, a 2.59-cm papillary predominant adenocarcinoma with no high-grade pattern in the right upper lobe(RUL) manifesting as a pure ground glass nodule(pGGN). All three model classified the lesion as low/intermediate-grade group; **(D)** Intermediate-grade, M, 61Y, T1b, a 1.73-cm acinar predominant adenocarcinoma with 3% high-grade patterns in the left upper lobe(LUL) manifesting as a sub-solid nodule(SSN). CRM and CRRM classified the lesion as low/intermediate-grade group, RM classified the lesion as high-grade group; **(E)** High-grade, M, 63Y, T1c, a 2.45-cm acinar predominant adenocarcinoma with 20% high-grade patterns in the right upper lobe(RUL) manifesting as a solid nodule (SN). RM and CRRM classified the lesion as high-grade group, CRM classified the lesion as low/intermediate-grade group; **(F)** High-grade, M, 73Y, T1c, a 2.85-cm solid predominant adenocarcinoma with 80% high-grade patterns in the right upper lobe(RUL) manifesting as a solid nodule (SN). RM and CRRM classified the lesion as high-grade group, CRM classified the lesion as low/intermediate-grade group.

The CT images were analyzed and recorded by two senior radiologists with more than ten years’ experience in respiratory system imaging diagnosis, and a consensus diagnosis was reached after discussion: a) lesions density, including pure ground glass nodule (pGGN), mixed ground glass nodule (mGGN), sub-solid nodule (SSN) and solid nodule (SN), where mGGN was defined as the ratio of the solid portion to the maximum diameter of the entire lesion<1/2, and SSN lesion was defined as the ratio of the solid part to the maximum diameter of the entire lesions≥1/2; b) long-axis and short-axis diameter of lesion; c) internal signs of the lesion; d) relationship between lesion and blood vessel; e) relationship between lesion and bronchus; f) lesion edge. The representative cases are shown in **Figure 2**.

### Statistical analysis

Statistical analysis used SPSS (version 26.0, https://www.ibm.com) and MedCalc software (version 20.104, https://www.medcalc.org). The normality of the data was tested using the Shapiro-Wilk normality test. The continuous variable with normal distribution was expressed as (mean ± SD), and the independent sample t-test was used for comparison between groups; the continuous variable with non-normal distribution was expressed as M (P25, P75), and the Mann-Whitney U test was used for comparison between groups; The chi-square test or Fisher’s exact test was used to compare categorical variable. ICC was used to evaluate the consistency of radiomic features, and ICC≥0.75 represented good repeatability. Logistic regression (LR) classifier was used to establish radiomics model (RM), clinical-radiological features model (CRM), and combined rad-score with radiological features model (CRRM). The area under the curve (AUC) of the receiver operating characteristic (ROC) curve and calibration curve were used to evaluate the performance and fitness of models. Delong test was used to compare AUC between models, and *p*<0.05 was considered statistically significant.

## Result

### Clinical and CT radiological features

The training set included 159 patients (130 low/intermediate-grade; 29 high-grade), the validation set included 69 patients (56 low/intermediate-grade; 13 high-grade) and the test set included 66 patients (49 low/intermediate-grade; 17 high-grade). The clinical and radiological features of patients are compared in **Table 1**.

**Table 1:** The clinical and CT radiological features of patients in the training, validation and test set.

Features	Training set (n =159)		*p*	Validation set (n=69)		*p*	Test set (n=66)		
	Low/intermediate-grade (n = 130)	high-grade (n = 29)		Low/intermediate-grade (n = 56)	high-grade (n = 13)		Low/intermediate-grade (n = 49)	high-grade (n = 17)	
Age (years)			0.038			0.632			
Mean ± SD/M (P25, P75)	60.00 (54.00, 66.00)	64.00 (58.70, 68.90)		62.07 ± 8.09	60.92 ± 6.01		60.18 ± 8.50	62.29 ± 8.56	0.382
Sex			0.111			0.473			0.404
Male	55 (42.31%)	17 (58.62%)		24 (42.86%)	7 (53.85%)		20 (40.82%)	5 (29.41%)	
Female	75 (57.69%)	12 (41.38%)		32 (57.14%)	6 (46.15%)		29 (59.18%)	12 (70.59%)	
Smoking history			0.806			0.101			0.131
No	97 (74.62%)	21 (72.41%)		45 (80.36%)	7 (53.85%)		44 (89.80%)	12 (70.59%)	
Yes	33 (25.38%)	8 (27.59%)		11 (19.64%)	6 (46.15%)		5 (10.20%)	5 (29.41%)	
Clinical stage			0.330			0.003			0.326
IA 1	7 (5.38%)	0 (0.00%)		2 (3.57%)	0 (0.00%)		2 (4.08%)	0 (0.00%)	
IA 2	75 (57.69%)	16 (55.17%)		37 (66.07%)	3 (23.08%)		27 (55.10%)	8 (47.06%)	
IA 3	39 (30.00%)	10 (34.48%)		14 (25.00%)	6 (46.15%)		13 (26.53%)	5 (29.41%)	
IB	9 (6.92%)	3 (10.34%)		3 (5.36%)	4 (30.77%)		7 (14.29%)	4 (23.53%)	
Long-axis diameter (cm)	1.76 (1.41, 2.19)	1.84 (1.38, 2.67)	0.402	1.62 (1.27, 2.15)	2.69 (2.00-3.68)	0.001	1.78 (1.43, 2.46)	2.07 (1.53, 2.76)	0.250
Short-axis diameter (cm)	1.37 (1.02, 1.75)	1.58 (1.12, 1.89)	0.124	1.26 (0.94, 1.58)	1.89 (1.34,2.33)	0.003	1.33 (0.99, 1.72)	1.76 (1.26, 2.03)	0.096
Lobe location			0.008			0.245			0.270
RUL	60 (46.15%)	5 (17.24%)		16 (28.57%)	6 (46.15%)		20 (40.82%)	5 (29.41%)	
RML	5 (3.85%)	2 (6.90%)		3 (5.36%)	2 (15.38%)		5 (10.20%)	1 (5.88%)	
RLL	26 (20.00%)	9 (31.03%)		14 (25.00%)	1 (7.69%)		8 (16.33%)	3 (17.65%)	
LUL	30 (23.08%)	6 (20.69%)		17 (30.36%)	2 (15.38%)		15 (30.61%)	5 (29.41%)	
LLL	9 (6.92%)	7 (24.14%)		6 (10.71%)	2 (15.38%)		1 (2.04%)	3 (17.65%)	
Nodule density			<0.001			<0.001			<0.001
pGGN	21 (16.15%)	0 (0.00%)		14 (25.00%)	0 (0.00%)		2 (4.08%)	0 (0.00%)	
mGGN	53 (40.77%)	4 (13.79%)		30 (53.57%)	2 (15.38%)		24 (48.98%)	1 (5.88%)	
SSN	30 (23.08%)	0 (0.00%)		7 (12.50%)	1 (7.69%)		14 (28.57%)	3 (17.65%)	
SN	26 (20.00%)	25 (86.21%)		5 (8.93%)	10 (76.92%)		9 (18.37%)	13 (76.47%)	
Margin			0.043			0.135			1.000
Circumscribed	128 (98.46%)	26 (89.66%)		53 (94.64%)	10 (76.92%)		49 (100.00%)	17 (100.00%)	
Speculated	2 (1.54%)	3 (10.34%)		3 (5.36%)	3 (23.08%)		0 (0.00%)	0 (0.00%)	
Pleural indentation			0.008			0.110			0.001
No	71 (54.62%)	8 (27.59%)		31 (55.36%)	4 (30.77%)		26 (53.06%)	1 (5.88%)	
Yes	59 (45.38%)	21 (72.41%)		25 (44.64%)	9 (69.23%)		23 (46.94%)	16 (94.12%)	
Lobulation			0.141			0.090			0.809
No	5 (3.85%)	0 (0.00%)		4 (7.14%)	0 (0.00%)		2 (4.08%)	1 (5.88%)	
Shallow lobulation	35 (26.92%)	4 (13.79%)		17 (30.36%)	1 (7.69%)		3 (6.12%)	0 (0.00%)	
Deep lobulation	90 (69.23%)	25 (86.21%)		35 (62.50%)	12 (92.31%)		44 (89.80%)	16 (94.12%)	
Spiculation			0.006			<0.001			0.009
No	62 (47.69%)	8 (27.59%)		37 (66.07%)	2 (15.38%)		16 (32.65%)	1 (5.88%)	
Short spiculation	13 (10.00%)	11 (37.93%)		4 (7.14%)	7 (53.85%)		29 (59.18%)	10 (58.82%)	
Long spiculation	55 (42.31%)	10 (34.48%)		15 (26.79%)	4 (30.77%)		4 (8.16%)	6 (35.29%)	
Bubblelike lucency			0.296			0.945			0.725
No	77 (59.23%)	15 (51.72%)		35 (62.50%)	8 (61.54%)		25 (51.02%)	11 (64.71%)	
<3mm	36 (27.69%)	6 (20.69%)		13 (23.21%)	3 (23.08%)		19 (38.78%)	2 (11.76%)	
>3mm	17 (13.08%)	8 (27.59%)		8 (14.29%)	2 (15.38%)		5 (10.20%)	4 (23.53%)	
Bronchovascular			<0.001			0.001			0.013
Irrelevant	0 (0.00%)	0 (0.00%)		0 (0.00%)	0 (0.00%)		1 (2.04%)	1 (5.88%)	
Attachment	17 (13.08%)	1 (3.45%)		2 (3.57%)	0 (0.00%)		2 (4.08%)	0 (0.00%)	
Through/Convergence	91 (70.00%)	11 (37.93%)		49 (87.50%)	4 (30.77%)		27 (55.10%)	2 (11.76%)	
Interruption	22 (16.92%)	17 (58.62%)		5 (8.93%)	9 (69.23%)		19 (38.78%)	14 (82.35%)	
Bronchial change			0.959			0.623			0.764
No	52 (40.00%)	14 (48.28%)		17 (30.36%)	8 (61.54%)		8 (16.33%)	5 (29.41%)	
Air bronchogram	38 (29.23%)	5 (17.24%)		23 (41.07%)	0 (0.00%)		19 (38.78%)	4 (23.53%)	
Thickened	19 (14.62%)	2 (6.90%)		13 (23.21%)	1 (7.69%)		6 (12.24%)	2 (11.76%)	
Stenosis/Interruption	21 (16.15%)	8 (27.58%)		3 (5.36%)	4 (30.77%)		16 (32.65%)	6 (35.29%)	
Micro-calcification			0.086			0.188			1.000
No	129 (99.23%)	27 (93.10%)		56 (100.00%)	12 (92.31%)		49 (100.00%)	17 (100.00%)	
Yes	1 (0.77%)	2 (6.90%)		0 (0.00%)	1 (7.69%)		0 (0.00%)	0 (0.00%)	
Lymphadenopathy			0.332			1.000			1.000
No	129 (99.23%)	28 (96.55%)		56 (100.00%)	13 (100.00%)		48 (97.96%)	17 (100.00%)	
Yes	1 (0.77%)	1 (3.45%)		0 (0.00%)	0 (0.00%)		1 (2.04%)	0 (0.00%)	

RUL, right upper lobe; RML, right middle lobe; RLL, right lower lobe; LUL, left upper lobe; LLL, left lower lobe; pGGN, pure ground glass nodule; mGGN, mixed ground glass nodule; SSN, sub-solid nodule; SN, solid nodule.

### Feature selection and radiomic model construction

**Figure 3:**
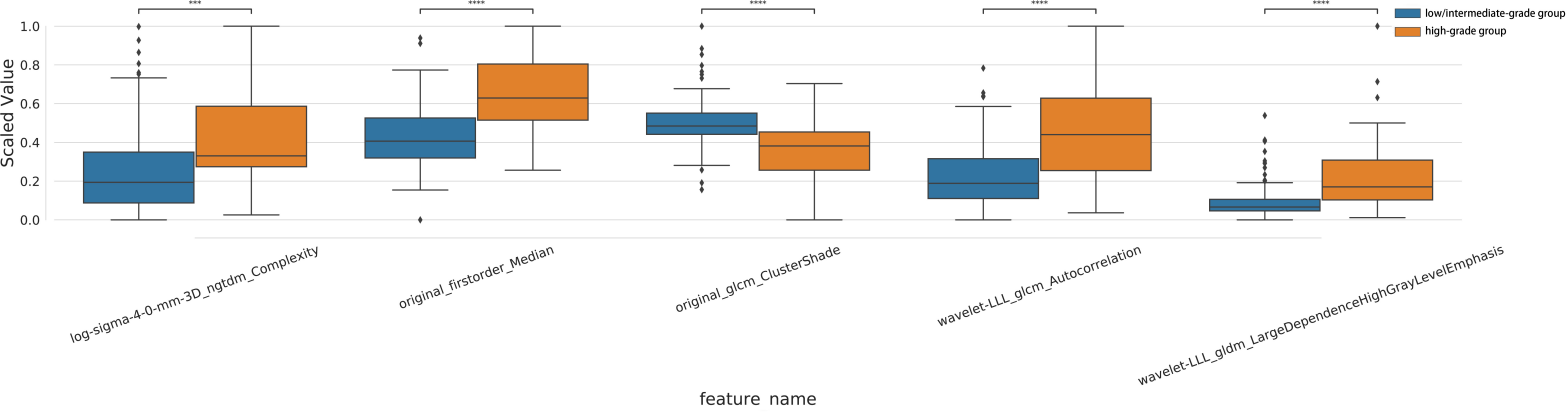
Intra-group distribution and inter-group analysis of the five optimal radiomic features in training set.

After a series of feature selection, five optimal radiomic features were obtained, including log-sigma-4-0-mm-3D_ngtdm_Complexity, original_firstorder_Median, original_glcm_ClusterShade, wavelet-LLL_glcm_Autocorrelation, wavelet-LLL_gldm_LargeDependenceHighGrayLevelEmphasis. The feature weighting coefficients were obtained by LASSO-LR. The weights and comparisons of each feature are shown in **Table 2** and **Figure 3**. In the training set, the above five radiomic features were used to construct RM using LR classifier to predict the pathological grade of lPA, and the radscore of each patient was calculated.

**Table 2:** Name and weighting coefficient of five optimal radiomic features.

Radiomic features	Weighting coefficient
log-sigma-4-0-mm-3D_ngtdm_Complexity	0.092813
original_firstorder_Median	0.147805
original_glcm_ClusterShade	-0.128176
wavelet-LLL_glcm_Autocorrelation	0.117018
wavelet-LLL_gldm_LargeDependenceHighGrayLevelEmphasis	0.126366

ngtdm, neighborhood gray-tone difference matrix; glcm, gray level co-occurrence matrix.

### Radiological features selection and clinical-radiological features model construction

The relationships between clinical-radiological features and IPA pathological grade in the training set were shown in **Table 3**. Univariate analysis showed statistically significant in seven variables between low/intermediate-grade and high-grade. Multivariate analysis showed pulmonary nodule density [odds ratio (OR) =1.165; 95% confidence interval (CI)=1.108-1.225; *p*<0.001], margin (OR=1.454; 95%CI=1.076-1.965; *p*=0.015), lesion location of LLL (OR=1.238; 95%CI= 1.038-1.476;*p*=0.018), which were independently associated with IPA pathological grade. The above three risk factors in the training set were used to construct CRM using LR classifier. The expression of the model was: Logit(P)=-0.263 + 0.153× nodule density +0.374 × margin +0.214 × lesion location (left lower lobe).

**Table 3:** Relationships between clinical-radiological features and IPA pathological grade in the training set.

characteristics	Univariate analysis		Multivariate analysis	
	OR (95% CI)	*p*	OR (95% CI)	*p*
Age (years)	1.007 (1.001-1.013) 1.001 1.001 1.001 1.001 )	0.034	NA	
Lobe location				
RUL	reference		reference	
RML	1.114(0.830-1.495)	0.471		
RLL	1.101(0.952-1.273)	0.195		
LUL	0.980(0.848-1.132)	0.782		
LLL	1.328(1.092-1.614)	0.005	1.238(1.038-1.476)	0.018
Nodule density	1.183(1.124-1.244)	<0.001	1.165(1.108-1.225)	<0.001
Margin	1.539(1.096-2.162)	0.013	1.454(1.076-1.965)	0.015
Pleural indentation	1.175(1.044-1.322)	0.007	NA	
Spiculation	1.151(1.061-1.250)	0.001	NA	
Bronchovascular	1.251(1.135-1.380)	<0.001	NA	

RUL, right upper lobe; RML, right middle lobe; RLL, right lower lobe; LUL, left upper lobe; LLL, left lower lobe.

NA, not applicable.

### Combined rad-score with radiological features model construction

**Figure 4:**
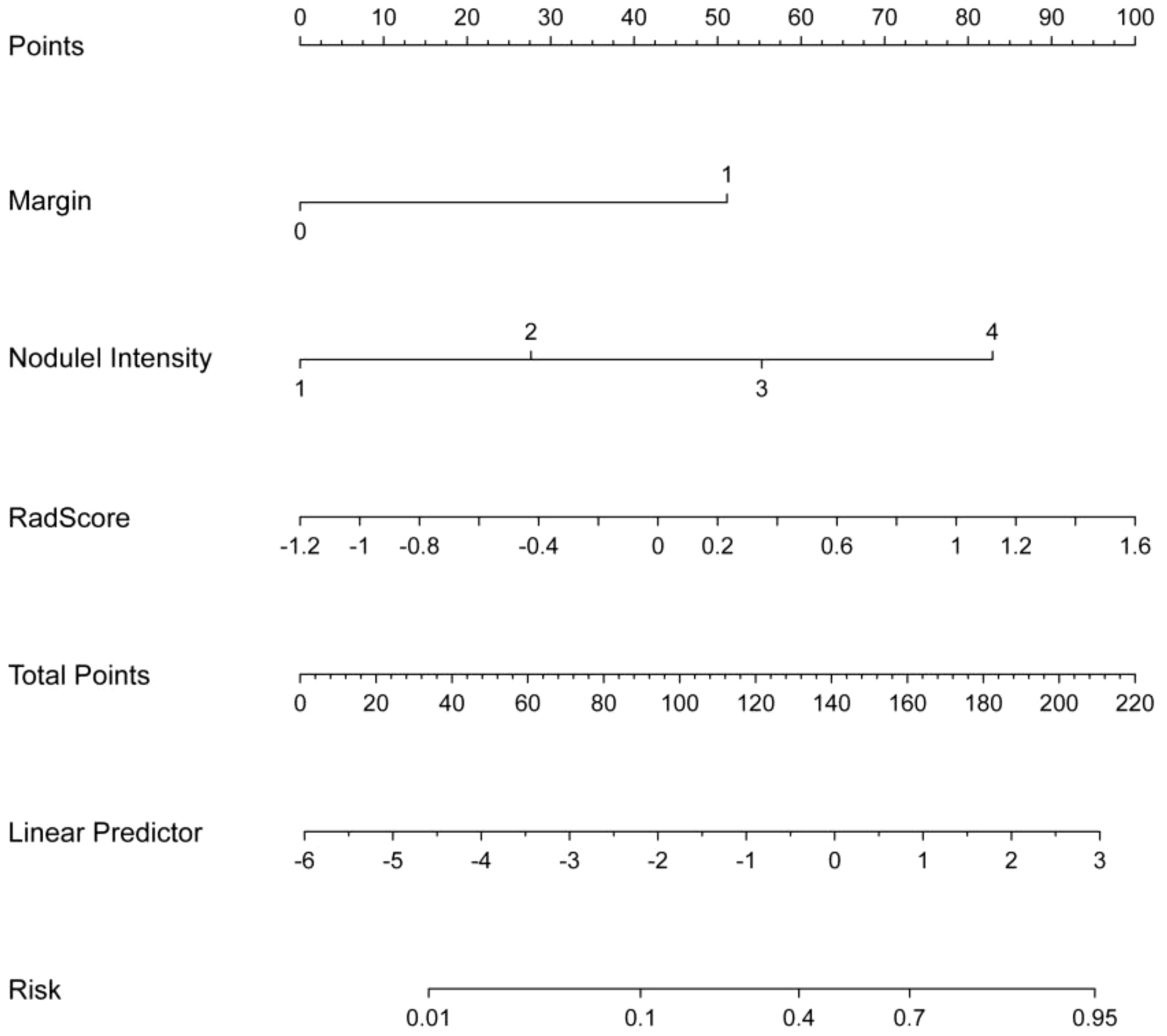
Nomogram of CRRM based on the training set.

The relationships between rad-score and radiological features and IPA pathological grade in the training set were shown in **Table 4**. Multivariate analysis revealed pulmonary nodule density (OR=1.079; 95% CI=1.012-1.149; *p*=0.019), margin (OR=1.387; 95% CI =1.037- 1.854; *p*=0.027), and rad-score (OR=1.335; 95% CI=1.164-1.532; *p*<0.001), which were independently associated with IPA pathological grade. The above three independent predictors in the training set were used to construct CRRM using LR classifier. The expression of the model was: Logit(P)=0.024 + 0.076× nodule density + 0.327× margin + 0.289×Radscore, and visualized CRRM by nomogram (**Figure 4**). And the pathological grade of patients can be predicted individually through the nomogram. The higher the calculated value, the greater the possibility of higher pathological grade of IPA.

**Table 4:** Relationships between rad-score and radiological features and IPA pathological grade in the training set.

characteristics	Multivariate analysis		
	OR	95%CI	*p*
Lobe location. LLL	NA		
nodule density	1.079	1.012- 1.149	0.019
Margin	1.387	1.037- 1.854	0.027
Radscore	1.335	1.164- 1.532	<0.001

LLL, left lower lobe.

NA, not applicable.

### Validation and evaluation of the model performance

**Figure 5:**
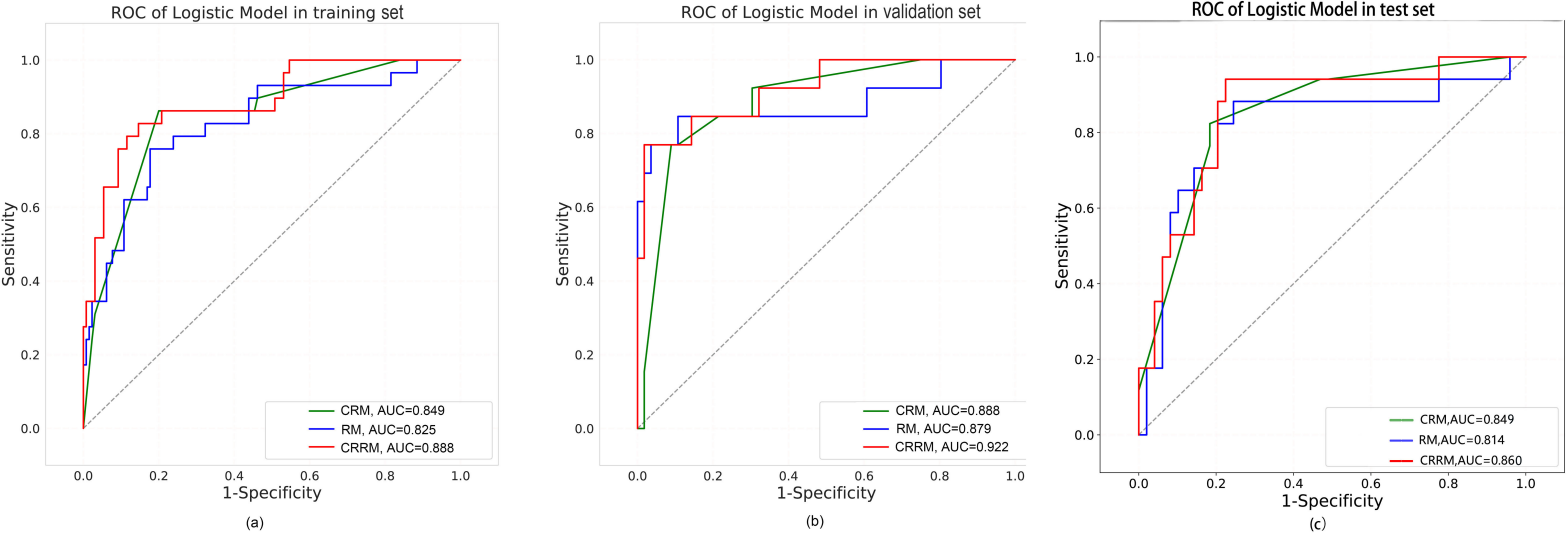
ROC curves for RM, CRM and CRRM in the training set **(A)**, validation set **(B)** and test set **(C)**.

**Figure 6:**
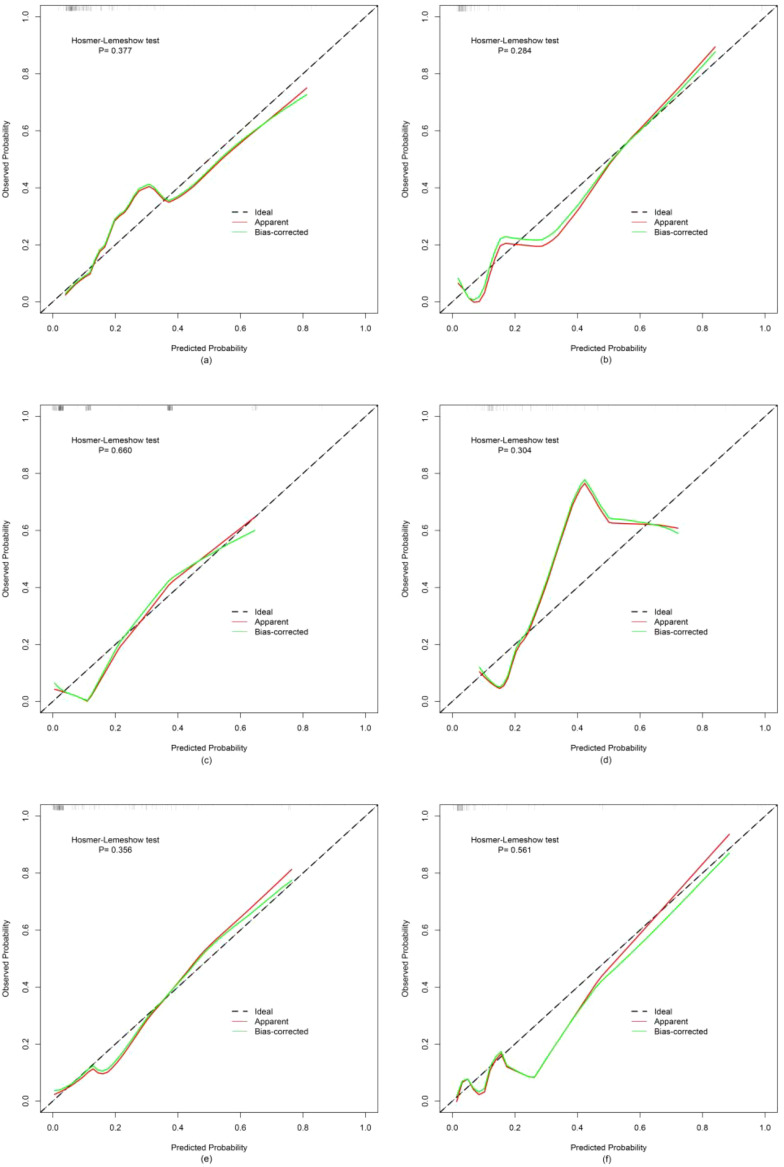
Calibration curves for RM, CRM and CRRM in the training set **(A, C, E)** and validation set **(B, D, F)**.

Finally, three models were constructed to predict pathological grade, including RM, CRM, and CRRM. The ROC curves, AUC, 95% CI-AUC, accuracy, sensitivity, and specificity for each model in the training, validation and test set are shown in **Figure 5**, **Table 5**. The AUC of CRRM was the highest in both training, validation and test set (AUC=0.888,95%CI=0.819-0.957;AUC=0.922,95%CI=0.835-1.000;AUC=0.860,95%CI=0.755-0.964), and there was statistically significant among the training set (Delong test: RM vs. CRRM, *p*= 0.028; CRM vs. CRRM, *p*=0.013). The AUC of CRM was slightly higher than RM in both training, validation and test set [AUC=0.849(95%CI=0.772 - 0.925) vs. AUC=0.825(95%CI=0.735 - 0.916); AUC=0.888(95% CI =0.794-0.982) vs. AUC=0.879(95% CI=0.734-1.000); AUC=0.849(95% CI =0.750-0.948) vs. AUC=0.814(95% CI=0.674-0.954)], the difference was not statistically significant (Delong Test, *p*>0.05). The calibration curve showed that the predicted probability of pathological grade by RM, CRM, and CRRM were highly consistent with the observed probability, as shown in **Figure 6**.

**Table 5:** Comparison between the training, validation and test set models.

Models	Sets	AUC	95%CI	Accuracy	Sensitivity	Specificity
RM	Training	0.825	0.735-0.916	80.5%	0.724	0.823
	Validation	0.879	0.734-1.000	87.0%	0.769	0.893
	Test	0.814	0.674-0.954	77.3%	0.882	0.735
CRM	Training	0.849	0.772-0.925	84.9%	0.310	0.969
	Validation	0.888	0.794-0.982	84.1%	0.308	0.964
	Test	0.849	0.750-0.948	77.3%	0.118	1.000
CRRM	Training	0.888	0.819-0.957	84.3%	0.793	0.854
	Validation	0.922	0.835-1.000	90.0%	0.769	0.929
	Test	0.860	0.755-0.964	78.8%	0.706	0.816

RM, radiomics model; CRM, clinical-radiological features model; CRRM, combined rad-score with radiological features model.

## Discussion

Our study showed that the AUCs for three models—radiomics model (RM), clinical-radiological model (CRM), and combined radiomics-radiological model (CRRM)—ranged from 0.825 to 0.888 in the training set, 0.879 to 0.922 in the validation set and 0.814 to 0.860 in the test set, all indicating well prediction performance. Notably, the AUC of CRRM was the highest across all sets, with AUC = 0.888 (95% CI = 0.819-0.957) in the training set, AUC = 0.922 (95% CI = 0.835-1.000) in the validation set, and AUC = 0.860 (95% CI = 0.755-0.964) in the test set. Furthermore, the nomogram based on the rad-score and radiological features, can be used as an intuitive and non-invasive tool for predicting the preoperative pathological grade of early IPA.

Because the sample size of preoperative needle biopsy is small, it cannot fully reflect all pathological pattern of IPA, so it usually cannot meet the pathological grade diagnosis of IPA. In addition, biopsy techniques may not be appropriate for small nodules. In contrast, preoperative CT-based radiomics model is not affected by sampling bias, it can reflect the overall characteristics of the nodule, offer more detailed differential grading information before surgery, facilitate histopathological diagnosis, and is non-invasive, making it particularly suitable for patients who are unable or unwilling to have surgery. Numerous studies have demonstrated that the presence of high-grade pattern is associated with poor prognosis, even if it is not the predominant subtype, it will increases the risk of lymph node metastasis and local recurrence (5, 6, 24). Therefore, lobectomy and systemic lymph node dissection should be considered for these patients with a high-grade pattern≥20% (25, 26). Additionally, previous studies have indicated that patients with high-grade pattern may benefit from adjuvant chemotherapy and improve disease-free survival (11, 13). Conversely, the lepidic predominant subtype is associated with the best prognosis, with low incidence of lymph node metastasis and rare recurrence. Jung et al. found that the median volume doubling time of the lepidic predominant subtype was over 1000 days, suggesting it may be more suitable for conservative surveillance (27). For the acinar/papillary predominant subtype, the prognosis is intermediate, with the possibility of positive surgical intervention and regular reexamination. Therefore, CT-based radiomics model can help determining the pathological grading of IPA, potentially guiding personalized treatment options, such as conservative surveillance, appropriate surgical approaches, or adjuvant chemotherapy.

Several previous studies have used radiomics to predict the pathological grade of pulmonary adenocarcinoma. Bae et al. predicted the pathological grading of 91 patients with stage I-II pulmonary adenocarcinoma using a dual-energy CT radiomic signature (28). Patients were classified into three grades according to the architectural grading system, the AUC of the prediction model was 0.9307, 0.8610, and 0.8394, respectively. Park et al. used CT radiomic signature to differentiate the predominant subtypes based on Sica’s grading system, achieving model AUCs of 0.892 and 0.895 in the training and validation sets, respectively (29). The studies mentioned above were based on the older pathological grading system. Currently, there are few radiomics studies focusing on the new pathological grading system. Tang et al. investigated multiparametric MRI-based radiomic signature for preoperative prediction of histological grade in patients with non-small cell lung cancer, demonstrating that the radiomics-clinical nomogram had the potential to distinguish histological grade in non-small cell lung cancer, with AUCs of 0.814 and 0.767 in the training and validation sets, respectively (30). Their results were comparable to ours, suggesting that radiomics can be used to non-invasively predict the pathological grade of IPA.

In our study, the rad-score of CRRM was calculated from the five optimal radiomic features. According to the feature definitions provided by the Image Biomarker Standardization Initiative (IBSI) (31), complexity is a Gaussian-transformed NGTDM feature that measures both macroscopic and local perivoxel changes. Autocorrelation is a wavelet-transformed GLCM feature that assesses image texture roughness. These two features reflect tumor heterogeneity. Our findings indicate that voxel complexity and image texture roughness are greater in high-grade IPA compared to low/intermediate-grade IPA, because the cell’s morphological characteristics of high-grade IPA under the microscope will be poorly differentiated, and the tumor heterogeneity will be higher. Median is a first-order feature that represents the median intensity of the gray level histogram. Cluster Shade is a GLCM feature that reflects the asymmetry of pixel distribution. Large Dependence High Gray Level Emphasis is a wavelet-transformed GLDM feature that indicates the image high-intensity voxels are more concentrated. These three features are closely related to the nodule density of the tumor. In our study, the median gray value and the concentration of high intensity voxels in high-grade IPA were found to be higher than those in low/intermediate-grade IPA, whereas the asymmetry was lower. This difference is primarily due to the fact that high-grade IPA are mainly manifested as SN on CT images, low/intermediate-grade IPA are mainly manifested as mGGN. Consequently, the mean asymmetry of entire SN was lower than that of nodules mixed ground-glass and solid density, while the median gray value and high-intensity voxel concentration were higher than mGGN.

In CRRM, two radiological features of pulmonary nodules density and margin were independent predictors. Our findings indicate that the nodule density is closely associated with the degree of tumor differentiation. Specifically, when the lesion is solid, the pathological grade of the tumor tends to be lower, which was consistent with the findings of Fujikawa et al (32). Low-grade IPA is characterized by slow growth, which serves as the pathological basis for the circumscribed tumor margin. Conversely, as tumor differentiation decreases, high-grade IPA may present as a solid nodule with a speculated margin. This phenomenon can be attributed to several factors: first, the tumor exhibits a crab-like growth pattern, infiltrating and interlacing with the normal lung parenchyma; second, there is a degree of inflammatory reaction in the peritumoral lung parenchyma; third, there is tumor thrombus formation in the peritumoral small blood vessels and lymphatic vessels. While the primary reason for these observations is the tumor’s growth pattern, the latter two factors serve as supplementary explanations.

This study has several limitations. First, it is a retrospective analysis, which may introduce bias in the data collection process. Specifically, our study only focused on patients with stage I IPA from a single medical center. It is important to note that we conducted our analysis using 500 random samples at a ratio of 7:3 and assessing the performance of the models through an independent test set. Despite these limitations, we believe that our retrospective study still holds potential application value. In further study, it is necessary to include patients with stage II-IV IPA and carry out multi-center cooperation to enhance the generalization and robustness of the models. Second, the regions of interest (ROIs) in this study were manually contoured by two radiologists concurrently, ensuring that the radiomic features had an intraclass correlation coefficient (ICC) of ≥0.75. Nonetheless, some degree of subjective error may still be present. Third, the clinical information included into the study was not comprehensive, lacking details such as blood tumor markers and driver mutations. Future multi-dimensional omics studies could enhance precision medicine for the diagnosis and treatment of non-small cell lung cancer. Finally, in addition to distinguishing the pathological grade of early-stage IPA, CT-based radiomic parameters can also differentiate between radiation pneumonitis and immune pneumonitis, as well as predict PD-L1 expression and CD8 expression levels. We will continue to carry out further research to explore other applications of CT radiomics in the thoracic oncology.

In conclusion, the models based on radiomics, clinical-radiological features, and radiomics combined with radiological features had well performance in predicting pathological grade of early-stage IPA, especially the combined model, which was expected to be used as a supplementary method for preoperative non-invasive evaluation.

## Data Availability

The raw data supporting the conclusions of this article will be made available by the authors, without undue reservation.
